# Low Luminance Visual Acuity and Low Luminance Deficit in Proliferative Diabetic Retinopathy

**DOI:** 10.3390/jcm10020358

**Published:** 2021-01-19

**Authors:** Eleni Karatsai, Piyali Sen, Sarega Gurudas, Sobha Sivaprasad

**Affiliations:** 1 NIHR Moorfields Biomedical Research Centre, London EC1V 2PD, UK; e.karatsai@nhs.net (E.K.); p.sen@nhs.net (P.S.); 2 Institute of Ophthalmology, University College, London EC1V 9EL, UK; sarega.gurudas.17@ucl.ac.uk

**Keywords:** best corrected visual acuity, low luminance visual acuity, proliferative diabetic retinopathy, pan-retinal photocoagulation, aflibercept, low luminance deficit

## Abstract

This study aimed to determine the relation of best corrected visual acuity (BCVA) and low luminance visual acuity (LLVA) in proliferative diabetic retinopathy (PDR) following treatment with either aflibercept or pan-retinal photocoagulation (PRP). The study was conducted as a post-hoc analysis of the CLARITY trial in which naïve and PRP treated PDR patients were randomised to receive either aflibercept or PRP. BCVA and LLVA were assessed at baseline and at week 52. Our analyses showed that the BCVA and LLVA correlate well in treatment naïve PDR with an average low luminance deficit of 11.79 Early Treatment Diabetic Retinopathy Score (ETDRS) letters. However, LLVA at lower levels of BCVA showed more variance. Post aflibercept therapy, the mean change in BCVA and LLVA at 52 weeks after aflibercept was +2.1 (SD 6.05) letters and +0.39 (SD 5.6) letters, respectively. Similarly, after PRP, it was −2.5 (SD 4.9) letters and −1.9 (SD 8.7) letters, respectively. When comparing treatment arms, BCVA change was found to be statistically significant (*p* < 0.001) whereas LLVA was not (*p* = 0.11). These findings show that LLVA does not respond as well as BCVA following any treatment for PDR, even though BCVA and LLVA both test foveal function.

## 1. Introduction

Proliferative diabetic retinopathy (PDR) is characterised by the development of retinal or optic nerve neo-vascularisation in people with diabetes. Left untreated, PDR can result in severe visual loss [1]. Pan-retinal photocoagulation (PRP) is an established treatment for this condition but it destroys the peripheral retina causing regression of the retinal and optic nerve neovascularization [2]. Repeated PRP sessions may be required to treat recurrence of neo-vascularisation and may be associated with visual function loss such as peripheral field loss, impaired dark adaptation and reduced contrast sensitivity [3]. Recently, repeated intravitreal injections of anti-vascular endothelial growth factor (anti-VEGF) agents have been shown to be as effective as PRP in a five year follow-up study [4]. These anti-VEGF agents modulate the disease process and cause regression of the neo-vascularisation, but the disease recurs when the therapy is stopped.

Both PRP and anti-VEGF agents stabilise visual acuity in PDR [4,5]. The CLARITY study showed that aflibercept, an anti-VEGF agent, resulted in superior visual outcome compared to PRP in eyes with treatment with naïve PDR or active PDR after initial PRP [5]. Aflibercept was injected every month for three months followed by pro-re-nata dosing to treat recurrences, reactivation or new onset neo-vascularisation over 52 weeks [6].

Visual acuity tests such as the Snellen and Early Treatment Diabetic Retinopathy Score (ETDRS) are established in the assessment of visual function [7]. However, these are tested on high contrast charts and are affected by light scatter and wavefront aberrations. Visual acuity is often not affected in PDR unless complications such as diabetic macular oedema and/or ischaemia, epiretinal membrane, vitreous haemorrhage or tractional changes involve the fovea.

Mesopic visual acuity testing uses reduced contrast and is more translatable to real-world tasks such as driving at night [8]. It has also been shown to correlate better with certain diseases than visual acuity [9,10]. Although mesopic visual acuity is also a foveal function, this pathway is more complex and less well understood. This pathway is influenced by both the photoreceptors and the post-receptoral pathway [11].

Hypoxia is the key driver of PDR. As retinopathy advances, the hypoxic stimulus from capillary drop-off causes the development of new blood vessels on the retina and/or the optic disc. As the rod photoreceptors require a significant amount of oxygen during dark adaptation, this imbalance between oxygen demand and supply may compromise the function of the rod photoreceptors. However, subjective impairment of night vision has been rarely reported by people with untreated PDR [12]. Cone photoreceptors are also affected in diabetic retinopathy [13]. It is unclear whether mesopic visual acuity may be a better test of visual function than photopic visual acuity in eyes with PDR.

Mesopic or low luminance visual acuity (LLVA) is measured by adding a neutral density (ND) filter to the refraction for best corrected visual acuity (BCVA) whilst keeping the vision chart and lighting conditions of the room stable so that luminance is reduced by 2.0 log units [14]. Alternatively, the vision chart can be dimmed by a ND to provide the same effect. The difference between BCVA and LLVA is the low luminance deficit (LLD). In clinical trials on geographic atrophy, LLD of 20 letters or worse is a prognostic indicator of disease progression. It is not known whether there is a dissociation between BCVA and LLVA in PDR.

It is also unknown whether LLD is uniformly affected in all PDR eyes or whether it is dependent on baseline BCVA. Similarly, a change in LLVA and LLD before and after PRP may reveal the impact of PRP despite unchanged BCVA.

In this study, we aimed to study the relation of LLVA and BCVA in PDR and to measure any differences in LLVA outcomes after treatment of PDR with PRP and anti-VEGF therapy.

The research questions included: (1) what is the correlation between LLVA and BCVA in treatment of naïve PDR patients? (2) Is the dissociation between BCVA and LLVA (BCVA-LLVA = LLD) constant or does it vary with BCVA or LLVA in treatment naïve PDR? (3) In treatment of naïve PDR patients, does the change in BCVA and LLVA with aflibercept therapy differ from those treated with PRP by 52 weeks? (4) Does LLD differ in each type of treatment? (5) In eyes, previously treated with PRP, does repeat PRP worsen LLVA more than BCVA over 52 weeks?

## 2. Materials and Methods

This study is a post-hoc analysis of fully anonymised data obtained from the CLARITY trial so a separate institutional review board approval was not required. The CLARITY trial compared aflibercept versus PRP for patients with either treatment naïve or active PDR post initial PRP. Each participant provided informed consent and the trial was approved by the National Research Ethics Committee Service London, South East (14/LO/0203) [5].

### 2.1. CLARITY Study Participants

Patients with Type 1 or 2 diabetes with treatment naïve PDR or active PDR post initial PRP with BCVA of 54 or more Early Diabetic Retinopathy Study (ETDRS) letters with sufficient media clarity were deemed eligible for the study. Key exclusion criteria included coexistent ocular pathology that can affect visual acuity, macular oedema, dense vitreous haemorrhage, fibrovascular proliferation or tractional retinal detachment.

### 2.2. Treatment Regimen

Patients were randomised to have either PRP or repeated intravitreal aflibercept 2 mg/0.05 mL. Patients randomised to the aflibercept arm received mandated the loading injections and were then re-treated as and when necessary based on the regression patterns of new vessels [6]. Participants in the PRP arm had PRP at baseline and were re-assessed every eight weeks and re-treated with PRP, if necessary.

### 2.3. Data Collection

In this post-hoc analysis, data on BCVA and LLVA collected in the trial were analysed to answer the research questions. In the trial, refraction was performed by certified optometrists and BCVA was recorded using an ETDRS chart tested at four metres. If a patient could not read 20 or more letters at four metres, the test was repeated at one metre. The LLVA was measured at baseline and at week 52 following the same steps as above, but a neutral density filter was held in front of the tested eye to decrease the luminance by 2.0 log units. LLD was defined as the difference in letters read under the two testing conditions (BCVA minus LLVA) letters.

### 2.4. Outcomes

We evaluated the relation between BCVA and LLVA in treatment naïve eyes at baseline. We then analysed whether there was a difference between BCVA and LLVA outcome following anti-VEGF or PRP at 52 weeks. We also examined whether LLVA and LLD worsened with repeated PRP.

### 2.5. Statistical Analysis

All study parameters are reported as a percentage for categorical, mean and standard deviation for continuous variables. Independent t–test or nonparametric Mann Whitney U test was used to compare between groups based on the normality of data. *p* values of <0.05 were considered statistically significant. Spearman Correlation coefficients were reported to show the rank association between BCVA and LLVA in various treatment subgroups reported with 95% CIs. Statistical analysis was performed using Microsoft Excel version 14.0 (2010 Microsoft Corporation) and Stata version 15.1 (Stata Corp 2017, Stata Statistical Software: Release 15, College Station, TX, USA: StataCorp LLC.).

## 3. Results

**Figure 1:**
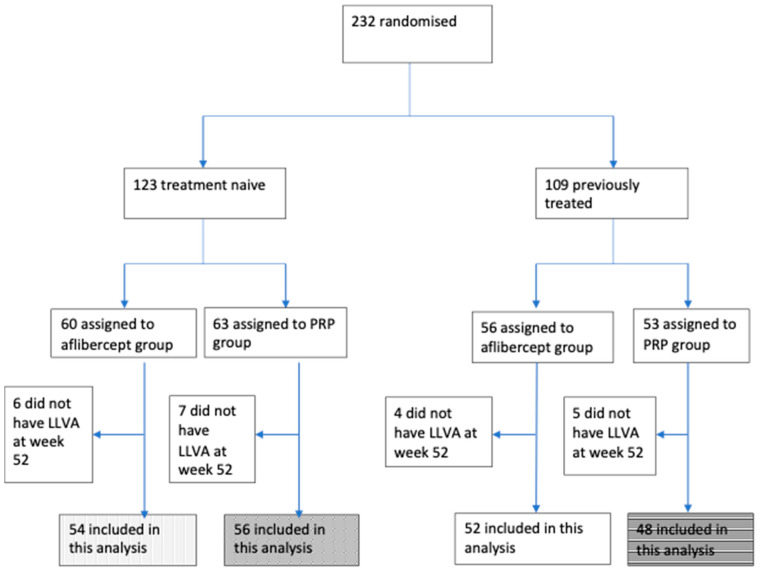
Flow chart of participants in this study. PRP: pan-retinal photocoagulation. LLVA: low luminance visual acuity.

The flow diagram (Figure 1) shows the CLARITY study groups that were evaluated in this post-hoc analysis. Treatment naïve groups with complete data (*n =* 110) were included to evaluate the correlation of BCVA and LLVA. This group was then assessed for the change in BCVA, LLVA and LLD following treatment with either aflibercept monotherapy (*n =* 54) or PRP (*n =* 56). The previously treated PRP group was assessed to understand the change in BCVA, LLVA and LLD and their correlations following repeat PRP (*n =* 48).

The flow chart shows the patients included in this study.

The baseline characteristics of all treatment naïve (*n =* 110) eyes with active PDR are described in Table 1. The average LLD was 11.79 (SD 6.08) letters, but eyes with better baseline BCVA had lower LLD than eyes with poorer baseline BCVA. This LLD pattern is related more to the variations in LLVA rather than BCVA. Participants with BCVA less than 84 letters (median or lower 50%) had SD LLVA of 8.78 compared to participants with BCVA greater than or equal to 84 letters where LLVA SD was 5.58 (*p* < 0.001; test for equality of variances). Similarly, in the upper 75% of BCVA (greater than 88 ETDRS letters) SD for LLVA was 4.19 compared to LLVA SD in the lower 75% which was 9.32 (*p* < 0.001). The same applied for the lower 25% (LLVA SD 10.4 vs. 7.19; *p*-value = 0.01).

The LLD was not influenced by any other parameters. There was no difference in LLD between eyes with only NVE (mean LLD 11.8 (SD 6.1) and eyes with NVD (mean 11.7 (SD 6.2) letters). Mean LLD was also similar in pseudo-phakic versus phakic eyes (mean 11.3 (SD 4.7) and 11.8 (SD 6.2)). There was also no age-related change in LLD. The mean LLD categorised by age groups <60 years, 60–69 years, 70–79 years and 80 years or older was 16 (SD 6.1), 14 (SD 7.7), 12.6 (SD 8.1) and 10.3 (SD 3.1), respectively.

**Figure 2:**
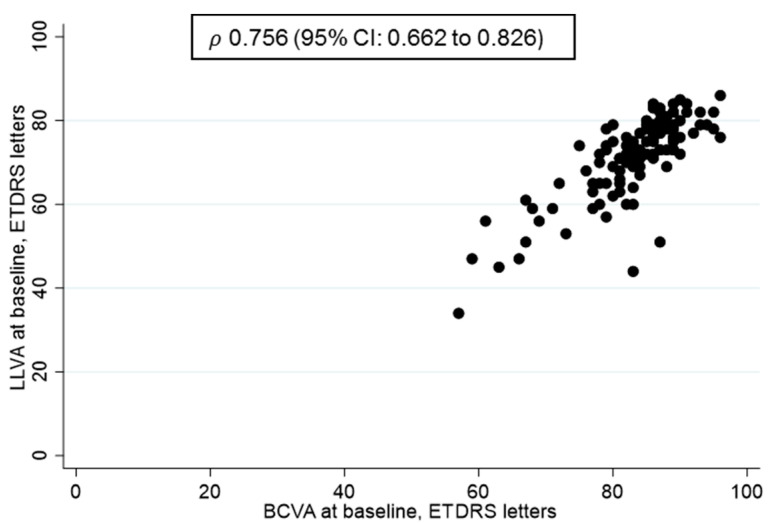
The relation between best corrected visual acuity (BCVA) and low luminance deficit (LLVA) in treatment naïve proliferative diabetic retinopathy (PDR) eyes. Spearman’s Correlation was 0.756 (95% CI: 0.662 to 0.826).

The correlations between BCVA and LLVA in treatment naïve eyes are shown in Figure 2. These visual functions are highly correlated although more variations were observed with LLVA in lower levels of BCVA.

### 3.1. Treatment Effect on BCVA and LLVA

In the aflibercept treated group (*n =* 54), the mean BCVA at baseline was 82.4 (SD 8.03) letters and mean LLVA was 71.7 (SD 10.20) letters. The mean LLD at baseline was 10.8 (SD 4.95) letters.

In the 56 treatment naïve eyes that underwent PRP, the mean BCVA and LLVA at baseline were 83.4 (SD 7.17) and 70.6 (SD 9.76) letters, respectively. Mean LLD at baseline was 12.8 (SD 6.89) letters.

In the aflibercept treated group, the mean BCVA at 52 weeks was 84.6 (SD 11.1) letters and mean LLVA was 72.1 (SD 11.9) letters. The mean LLD was 12.5 (SD 5) letters. The mean change in BCVA at 52 weeks after treatment with aflibercept was +2.1 (SD 6.05) letters and the mean change in LLVA was +0.39 (SD 5.6) letters. The mean change in LLD after aflibercept was 1.72(SD 5.83).

56 treatment naïve eyes underwent PRP and were also followed up for 52 weeks. Mean BCVA and LLVA at 52 weeks were 80.9 (SD 8.6) and 68.8 (SD 11.7) letters respectively. Mean LLD at 52 weeks was 12.1 (SD 6.7) letters. The mean change in BCVA from baseline at 52 weeks after PRP was −2.5 (SD 4.9) letters and the mean change in LLVA was −1.9 (SD 8.7) letters. The mean change in LLD following PRP was −0.64 (SD 7.39).

**Figure 3:**
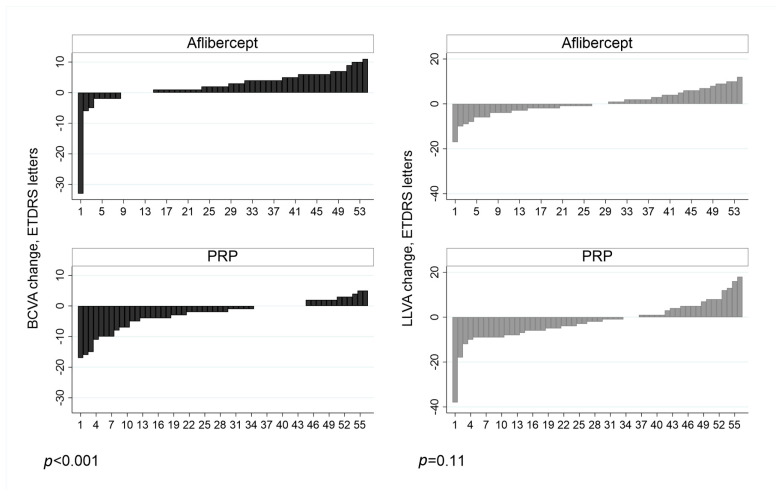
Shows the participants’ level of change in best corrected visual acuity (BCVA) and low luminance visual (LLVA) in treatment naïve patients at 52 weeks.

The individual patient-level response to treatment with aflibercept and PRP in treatment-naïve eyes is shown in Figure 3. When we compared the response between treatment arms, BCVA change was found to be statistically significant (*p* < 0.001) but LLVA was not (*p* = 0.11). The mean change in LLD between treatment arms in treatment naïve eyes did not reach statistical significance (*p* = 0.06; independent t-test).

Table 2 shows the categorical distribution of LLD in both treatment arms. Most participants in both arms were within ±5 letters of the mean difference of 12 letters observed before treatment. The proportion of participants with LLD > mean + 2SD in the aflibercept arm was 4 (7.41%) and PRP was 3 (5.36%).

### 3.2. Impact of Repeat PRP on BCVA, LLVA and LLD

The mean baseline BCVA of the 48 participants who had repeat PRP in the study was 80.8 (SD 7.5) letters and mean LLVA was 67.3 (SD 8.3) letters. Mean BCVA at week 52 was 77.9 (SD 10.2) letters and mean LLVA was 63.1 (SD 13.7) letters, whilst mean LLD was 14.8 (SD 6.9) letters. The mean change in BCVA from baseline at 52 weeks after repeat PRP was −3.6 (SD 8.8) letters and the mean change in LLVA was −4.77 (SD 11.99) letters. There was no significant difference in LLD in this group between baseline and after repeat PRP at 52 weeks.

### 3.3. Correlation between Mean Change in BCVA and LLVA in the Three Treatment Groups

**Figure 4:**
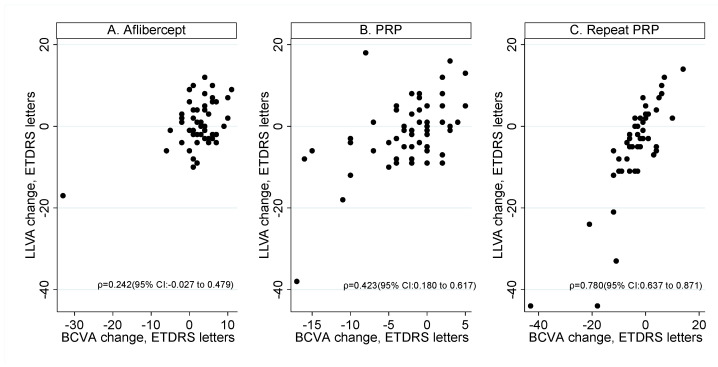
The correlation between change in best corrected visual acuity (BCVA) and low luminance visual acuity (LLVA) was not significant in the (**A**) aflibercept arm; (**B**) Panretinal photocoagulation (PRP) in treatment naïve eyes and (**C**) Repeat PRP.

Figure 4 shows the correlation of mean change in BCVA and mean change in LLVA at 52 weeks in the treatment naïve aflibercept arm, in the treatment naïve PRP arm and in the repeat PRP arm. There was a statistically significant correlation between change in BCVA and change in LLVA in treatment naïve eyes receiving PRP and eyes that had repeat PRP (*p* = 0.001, *p* < 0.001 for treatment naïve and repeat PRP eyes). The Spearman correlation coefficient for aflibercept eyes was not statistically significant (*p* = 0.073).

Spearman’s correlation coefficients (*ρ*) presented with 95% CIs. Removing the single observation with high drop in BCVA and LLVA in A) Aflibercept, Spearman’s correlation coefficient reduces to 0.198 (95% CI: −0.076 to 0.445) on 53 observations.

Despite the correlations shown in Figure 4, eyes with smaller changes in BCVA than the median showed more variability in LLVA change compared to those who experienced higher gains in BCVA. For example, for repeat PRP eyes, the SD of LLVA letter change in eyes with less than -3 BCVA letter change (median or 50%) was 6.07; and the SD of LLVA change in eyes with more than or equal to −3 letter BCVA change was 13.43 (*p* < 0.001, test for equality of variances). For treatment naïve eyes that had PRP, the SD in eyes with less than −2 (median or 50%) letter BCVA change was 10.43, whereas eyes with greater than or equal to −2 letter BCVA change was 6.64 (*p* = 0.02, test for equality of variances).

For treatment naïve eyes that had aflibercept, SD of LLVA letter change in eyes with less than 2 BCVA letter change was 6.11, and SD in eyes with greater than or equal to 2 letter BCVA change was 4.99 (*p* = 0.2960; test for equality of variances).

## 4. Discussion

In this post-hoc analysis of the CLARITY trial, we found that LLVA in treatment naïve eyes is on average two lines lower than BCVA when all other variables are kept constant. Simplistically, these observations can be explained by the fact that both BCVA and LLVA are foveal cone mediated visual functions, thus the linear difference is due to the decrease in luminance. However, the mesopic pathway is more complex and we observed that, although these visual functions are highly correlated, the difference between BCVA and LLVA (LLD) is more marked in lower levels of BCVA and this is driven by a more variable LLVA at lower levels of BCVA. Stockman et al. showed that mesopic vision is governed by several factors such as rod-cone interactions, their retinal distributions, their integrated responses and the post-receptoral pathways governing the rod and cone signals [11]. This dissociation between BCVA and LLVA at lower levels of BCVA suggests that the post-receptoral pathway may be more vulnerable than BCVA to neuronal and/or ischaemic changes in PDR. A faster but less sensitive rod pathway is also active at mesopic levels.

We also showed that although the change in BCVA was superior in the aflibercept arm compared to PRP, the changes in LLVA were not significant between these two treatment arms in treatment naïve eyes. These findings show that the mesopic pathway is likely to be slower to recover. We also showed that changes in BCVA did not correlate with changes in LLVA. Although the correlations were significant in the PRP arms, the change in LLVA in lower levels of change in BCVA are also more variable compared to those who had higher gains in BCVA. These substantiate the above findings that the vulnerable mesopic pathway seems to have a predictable response to either aflibercept or PRP. As our study duration was for only 52 weeks, these findings also suggest that the LLVA pathway is unlikely to show rapid responses if used as a clinical trial outcome.

When we compare our observations to results of LLVA in studies on intermediate age related macular degeneration (AMD) and geographic atrophy, we can postulate a few points. Firstly, Sunness et al. found that LLD worsens with worsening BCVA [15]. They also showed that the LLD in eyes with geographic atrophy with good BCVA was about 4.6 lines, compared to those with drusen only which showed an average LLD of 2.2 lines. Therefore, in addition to BCVA, retinal atrophy may have affected the mesopic pathway (LLVA) more profoundly than BCVA.

Other studies in AMD also substantiate these findings. Wu et al. classified AMD severity levels into six groups and reported that LLD was significantly different only in the non-foveal geographic atrophy (GA) group [16]. Furthermore, in a cross-sectional study, Cocce et al. showed that there are no differences in BCVA, LLVA and LLD between control, early and intermediate AMD, as the BCVA eligibility criteria of the study was Snellen 20/50 or better and the mean BCVA in each group was more than 80 letters [17]. Another study that evaluated the factors that determined LLD in AMD reported that LLD is affected by age, BCVA, AMD severity, presence of reticular pseudo-drusen and sub-foveal choroidal thinning in AMD patients [18]. Of these factors, only age and BCVA apply to this study but we found no age related change of LLD in our study cohort. In our study, only BCVA was related to LLVA. Our baseline inclusion criteria were limited to 54 ETDRS Letters (Snellen 20/80) or better. Similar dissociations between LLVA and BCVA have also been observed in central serous chorioretinopathy and macular telangiectasia type 2 when compared to controls [9,19]. These observations suggest that LLVA and LLD may not be disease specific changes and the changes may be predominantly determined by the integrity of the mesopic pathway.

Clinical trials on interventions in geographic atrophy have shown that the average LLD in geographic atrophy trials was >20 letters while for PDR it is about 12 letters [20,21,22,23]. In addition, an LLD of 20 or more letters is a risk factor of disease progression [22]. However, in PDR, our study shows that an LLD of 20 or more letters is a very rare event. Therefore, such a clinical trial outcome is not feasible in PDR. Moreover, our study shows that the mean LLVA and LLD changes post-PRP or aflibercept are minimal and may not be appropriate to be used as clinical outcome measures for future PDR trials.

The strengths of our study are the large sample size, the fact that the BCVA and LLVA measurements were protocol driven assessments and the retention rate at 52 weeks was high. To our knowledge, this is the first study on the effects of PDR and its treatment on LLVA.

The limitation of our study was that we did not incorporate Optical coherence tomography angiography (OCT-A) measurements, so the effect of diabetic macular ischaemia on BCVA and LLVA cannot be directly derived from this analysis. As the correlation of BCVA and size of foveal avascular zone is low to moderate, BCVA is not an ideal outcome measure for trials on diabetic macular ischaemia. We believe that studies on the effects of increasing macular ischaemia on LLVA need to be done to assess the rate of deterioration of LLVA when compared to BCVA in this condition. Our study cohort also did not include people with poor visual acuity as our BCVA cut-off was 54 letters to avoid eyes with no potential of BCVA improvement. Future research on LLVA in eyes with macular ischaemia confirmed by optical coherence tomography angiography is necessary to confirm or refute our postulation that the post-receptoral pathway may be more affected than the foveal cones in PDR and in diabetic macular ischaemia.

## Figures and Tables

**Table 1:** Demographic characteristics of treatment naïve eyes at baseline.

Baseline	Treatment Naïve (*n =* 110)	Repeated PRP Group (*n =* 48)
Age Mean ± SD	54.37 (14.56)	57.71 (12.04)
Age categories	N (%)	
30–50 years	50 (45.45%)	11 (22.92)
51–70 years	45 (40.91%)	30 (62.5)
71–80 years	15 (13.64%)	7 (14.58)
Male	67 (60.91%)	34 (70.83)
Female	43 (39.09%)	14 (29.17)
T1DM	55 (50.00%)	21 (43.75)
T2DM	55 (50.00%)	27 (56.25)
Eyes with NVD+/−NVE	36 (32.73%)	19 (39.58%)
Eyes with NVE only	72 (65.45%)	28 (58.33%)
Pseudo-phakic	12 (10.91%)	6 (12.5)
Phakic	98 (89.09%)	42 (87.5)
Mean best corrected visual acuity (BCVA) (SD)	82.94 (SD 7.59)	81.48 (7.65)
Median BCVA (IQR)	84 (80–88)	82 (77–86.75)
Mean low luminance visual acuity (LLVA) (SD)	71.15 (SD 9.94)	67.88 (7.52)
Median LLVA (IQR)	73 (66–78)	68 (64.25–73)
Mean low luminance deficit (LLD) (SD)	11.79 (SD 6.08)	13.60(5.51)
Median LLD (IQR)	11 (7–15)	14 (10–16.75)
LLD 1–5 letters	13 (11.81)	3(6.25%)
LLD 6–10 letters	38 (34.55%)	11(22.92%)
LLD 11–14 letters	30 (27.27%)	12(25.00%)
LLD 15–19 letters	21 (19.09%)	17(35.42%)
LLD 20 or more letters	8 (7.27%)	5(10.42%)
Proportion > mean + 2SD	2 (1.82%)	2(4.17%)

SD: standard deviation; DM: diabetes mellitus; NVD: neovascularization of the disc; NVE: neovascularization elsewhere; IQR: interquartile range; LLVA: low luminance visual acuity; LLD: low luminance deficit.

**Table 2:** LLD difference between treatment naive arms at 52 weeks.

	Aflibercept (*n =* 54)	PRP (*n =* 56)	*p*-Value
Mean LLD (SD)	12.48 (4.96)	12.14 (6.69)	0.76 ^a^
LLD 1–5 letters	1 (1.9%)	9 (16.1%)	0.07 ^b^
LLD 6–10 letters	22 (40.7%)	14 (25%)	
LLD 11–14 letters	18 (33.3%)	19 (33.9%)	
LLD 15–19 letters	8 (14.8%)	7 (12.5%)	
LLD 20 or more letters	5 (9.3%)	7 (12.5%)	

^a^ independent sample *t*-test; ^b^ Fisher’s exact test.

## Data Availability

The authors are prepared to share their data with other researchers.
